# Psychosocial factors associated with becoming a young father in Finland: a nationwide longitudinal study

**DOI:** 10.1186/1471-2458-12-560

**Published:** 2012-07-27

**Authors:** Venla Lehti, Andre Sourander, Lauri Sillanmäki, Hans Helenius, Tuula Tamminen, Kirsti Kumpulainen, Fredrik Almqvist

**Affiliations:** 1 Department of Child Psychiatry, University of Turku, Itäinen Pitkäkatu 1/Varia, Turku, 20014, Finland; 2 Center for Child and Adolescent Mental Health, North Norway (RBUP), University of Tromsø, 9037, Breivika, Norway; 3 Department of Biostatistics, University of Turku, Lemminkäisenkatu 1, 20014, Turku, Finland; 4 Department of Child Psychiatry, Medical School, University of Tampere, Tampere, 33014, Finland; 5 Department of Child Psychiatry, University of Eastern Finland, Alava hospital, P.O. Box 1777, 70211, Kuopio, Finland; 6 Department of Child Psychiatry, University of Helsinki, the Hospital of Children & Adolescents, Lastenlinnantie 2, 00250, Helsinki, Finland

**Keywords:** Young father, Childhood, Adolescence, Risk factor, Epidemiology

## Abstract

**Background:**

Little is known about the characteristics of boys who become fathers at young age. Some studies have suggested that antisocial adolescents are more likely to be young fathers. The aim of this study was to examine the associations of psychosocial factors in childhood with becoming a young father, and to assess if they are independent of criminal behavior in adolescence.

**Methods:**

The baseline assessment in 1989 included 2,946 boys born in 1981. Information about psychiatric symptoms at age eight was collected with Rutter questionnaires from parents and teachers and with the Child Depression Inventory from the children themselves. Data on criminal offenses at age 16–20 was collected from a police register. Register-based follow-up data on becoming a father under the age of 22 was available for 2,721 boys.

**Results:**

The factors measured at age eight, which were associated with becoming a young father independently of adolescent criminality, were conduct problems, being born to a young father and having a mother with a low educational level. Having repeatedly committed criminal offences in adolescence was associated with becoming a young father independently of psychosocial factors in childhood.

**Conclusions:**

Antisocial tendencies both in childhood and adolescence are associated with becoming a young father. They should be taken into consideration when designing preventive or supportive interventions.

## Background

There is no “right age” of becoming a parent, but it is evident that parents of various ages may have different kind of background and different needs. Teenage mothers have been studied from different perspectives, but little focus has been on boys who become fathers at early age [1]. It has been estimated that 5.5–17% of men, depending on their ethnic background, become fathers under the age of 20 in the USA [2] and 14% of men become fathers under the age of 22 in the UK [3]. The different age limit in these studies illustrates that the definition of “young father” or “early fatherhood” is culture-bound. The definition used in the study conducted in the UK fits better the prevailing norms in the Finnish society. Under the age of 22 most Finnish boys are still studying or performing the compulsory military service, which can be considered an important milestone in the transition to adulthood. Becoming a father at that age is likely to be exceptional, but no studies on this topic have been conducted.

Some studies have shown that young fathers have an increased risk of social exclusion, low income, chronic illnesses and low life satisfaction [4,5]. It is probable, however, that these problems are largely explained by the pre-existing factors of boys who become young fathers [4]. Identifying these factors is crucial for the better understanding of the pathways that lead to being a young father, and if needed, for providing adequate support for young fathers and their families. It has been suggested that the likelihood of becoming a young father is increased for those who have low family socioeconomic status [3,6-8], young parents [7,9] and low academic achievement [3,6-8,10,11], live in a disadvantaged neighborhood [10], and are clients of child welfare services [12]. The results concerning externalizing psychiatric symptoms are conflicting. Some studies have shown that conduct problems [13,14], aggressiveness [15,16], delinquency [10,17] and substance use [9,11] are independently associated with later becoming a young father. In other studies, the association between externalizing problems and young fatherhood has been explained by factors such as academic performance or family socioeconomic status [7,8]. There are also studies which have not shown an association between externalizing problems [9,10] or drug use [10] and becoming a young father even in unadjusted analyses. Studies on internalizing symptoms are fewer and focus almost exclusively on depression, but their results are more consistent; to our knowledge, no independent association with becoming a young father has been shown [8-10,14,18].

There are some methodological limitations, which decrease the generalisability of the results and complicate the comparison between different studies. Most previous longitudinal studies are based on samples which are poorly representative of the general population, because they are selected from small areas and often from socially disadvantaged populations. They are also rather small in sample size. There are a couple of exceptions. Two large British cohorts from the 1940’s and 50’s are nationally representative. However, the study based on the National Survey of Health and Development [13] includes only teacher reports of conduct problems in adolescence, and those based on the National Child Development Study include only a combined measure of externalizing and internalizing problems [3] or a measure of aggression [16]. A study from New Zealand [18] is also based on a sample which is not selected from a particularly high-risk area, but is restricted to one urban region and includes fewer than 500 males. In many studies, the baseline assessment has been conducted in adolescence and thus the follow-up time is short [8-11,13,18].

This study aims to overcome the limitations of previous studies. This is also one of the first studies on becoming a young father, which has been conducted in a country other than the USA, UK or New Zealand. The study is based on a large, nationally representative sample. Childhood factors have been assessed by three informants as early as at age eight. The aim of the study is to examine the association of psychosocial factors in childhood with becoming a father under the age of 22. A further aim is to study whether the association between adolescent antisocial behavior and becoming a young father found elsewhere is found in the Finnish context, and whether it explains the possible association between the childhood risk factors and becoming a young father.

## Methods

The study is part of the “Finnish 1981 Birth Cohort Study”, a multicenter child psychiatric study in Finland. Informed consent was obtained from the children's parents at baseline. The research plan was approved by the Ethics Committee of the Intermunicipal Hospital District of Southwest Finland.

### Participants

The original study sample was drawn from the total population of Finnish children born during 1981 (n = 60,007). It consisted of 6,017 children, representing 10% of the basic population. A representative sample of all communities was chosen from each university hospital district in Finland. They were selected according to their degree of urbanization: urban, sub-urban and rural. In small communities all children born in 1981 were selected. In larger cities, a representative subsample was drawn from all the school districts. Of the selected 6,017 children, 5,813 (96.6%) took part in the study in 1989. The number of male participants was 2,946. The sample was nationally representative. Sociodemographic characteristics of the participants were shown to correspond very closely to figures in national statistics. The design and the participants of the baseline assessment have been presented previously [19]. The information on adolescent antisocial behavior was collected from the Finnish National Police Register. To combine the information on risk factors with the outcome data, the boys in the cohort were identified by linking their personal identification number with the population register data. Of the 2,946 boys studied at baseline, information about becoming a father was available for 2,721 (92.4%).

### Measures at age eight

The children completed the questionnaires in the classroom. The researchers visited each school and met the teachers involved. The teachers sent the parent questionnaires via the child to the parents and the parents returned them in a sealed envelope to the teachers.

#### Psychiatric symptoms

Information on psychiatric symptoms at age eight was collected from the parents, the teachers and the children themselves. The parents were asked to complete the Rutter Scale, Parent version, which consists of 31 items [20], and the teachers were asked to complete the Rutter Scale, Teacher version [21], with 26 items. The answers are rated on a scale of 0–2. The range of total scores for the parent version is 0–62 points and for the teacher version 0–52 points. Both include three subscales: antisocial, hyperkinetic, and neuroticism. These are later in the text referred to as conduct problems, hyperactive problems, and emotional problems. In this study, summed psychopathology scores from teacher and parent subscales were used. All variables were used as continuous variables.

The children completed the Children's Depression Inventory (CDI), a 27-item self-report measure for children's depressive symptomatology [22]. The range of scores for each item is 0–2 and the total scores range from 0 to 54. The question concerning suicide was excluded for ethical reasons, so the version of the CDI used in this study consisted of 26 questions, with the total score ranging from 0 to 52 points. The variable was used as continuous.

#### Family background

Parents gave information on the family structure and on their own age and education. If the boy lived with two biological parents, the family structure was defined as intact. Mother’s and father’s educational level was measured by their completion of upper secondary school. Not completing upper secondary school was referred to as a low educational level. Parental age at the participating child’s birth was calculated through the parents’ own birth year. Parents born in 1959 or later were referred to as young parents since they had been under 23 years old when they became parents in 1981. Those born in 1958 or earlier were referred to as old parents.

#### School performance

Teachers were asked about the child’s school performance by giving them three alternatives: The student is 1 = above average, 2 = average or 3 = below average. In this study, the alternatives 1 and 2 were combined so that the “below average” students were compared with those whose level was “average or above”.

### Antisocial behavior in adolescence

Assessment of antisocial behavior in adolescence was based on data on criminal offences. It was collected from the Finnish National Police Register, which is a nationwide electronic database kept by the administration of the Finnish Police since 1997. It includes all the incidents identified by the police except warnings or admonitions and municipal parking fines. Minor traffic offenses such as mild speeding or not using seat belt are registered, but were not included in this study. All other offenses such as major traffic offenses, property offenses and personal offenses were included. The incidents included in this study had been registered during the years 1998–2001 when the boys were 16 to 20 years old. Four categories were used: no offenses, one or two offenses, three to five offenses and more than five offenses.

### Outcome

Information about the age when the boys had become fathers for the first time was collected from the Finnish Population Information System, which is maintained by the Population Register Center and local register offices. It contains basic information such as name, personal identity code, address, and family relations, of Finnish citizens and foreign citizens residing permanently in Finland. If a child’s parents are married, the husband of the mother is automatically registered as the father. In other cases such as in cohabitating relationships the child welfare officer of the municipality establishes the paternity. The officer contacts the unmarried mother and collects information needed for the establishment. Free paternity testing can be proposed by the child welfare officer or required by the parents. The alleged father usually acknowledges his paternity. If he does not do it voluntarily, the officer can take the case to the district court for legal proceedings. The information collected for this study included the dates of birth for the first children born to the boys in our sample. The follow-up ended when the boys turned 22 years.

### Statistical analysis

Associations between psychosocial risk factors and becoming a young father were quantified by calculating odds ratios with 95% confidence intervals using logistic regression analysis. Psychiatric symptoms were measured as continuous variables. Rutter scores given by a teacher and a parent were summed in all analyses. The odds ratios for continuous variables were calculated for the change in one standard deviation unit (SD). Other variables were only used as categorical ones. First single predictor analyses were conducted for childhood variables. The variables which remained significant at level p < 0.1 were selected to the Multipredictor model 1. The variables which remained significant at level p < 0.1 in the Multipredictor model 1 were included in the Multipredictor model 2 with the variable on adolescent criminality. All analyses were conducted with a binary response: becoming a father under the age of 22 or not. Statistical computations were conducted with SAS System for Windows, release 9.2/2008.

## Results

There were 143 boys (5.3% of the sample) who had become fathers by the age of 22. The youngest fathers were 16 years old. There were only 53 boys (1.9% of the sample), who had become fathers under the age of 20. The distribution of categorical variables is shown in Table 1.

**Table 1:** Distribution of categorical explanatory variables

	**n**	**%**
Age of the boy’s mother		
Old	2225	93.6
Young	151	6.4
Age of the boy’s father		
Old	2269	93.8
Young	149	6.2
Educational level of the boy’s mother		
High	760	29.8
Low	1789	70.2
Educational level of the boy’s father		
High	509	21.7
Low	1833	78.3
Family structure		
Intact	2182	83.3
Non-intact	436	16.7
School performance		
Average or above	2241	84.1
Below average	425	15.9
Police register information*		
No criminal offenses	2101	77.2
1-2 offenses	401	14.7
3-5 offenses	108	4.0
>5 offenses	111	4.1

Table 2 includes the childhood variables, which were significantly (p < 0.05) associated with becoming a young father in the single predictor analysis. These were mother’s as well as father’s young age and their low educational level, parent and teacher report of conduct problems and hyperactivity, and self-report of depressive symptoms. Table 2 also shows the single predictor analysis for the adolescent antisocial behavior, which was significantly (p < 0.001) associated with becoming a young father. The association was strongest for the category more than five offenses. It was not significant for the category three to five offenses.

**Table 2:** Logistic regression analyses with a binary response (becoming a father < 22 years or not)

	**Total**	**Young fathers**	**Single predictor**		**Multipredictor model 1**		**Multipredictor model 2**	
	**n**	**%**	**OR (95% CI)**	**p**	**OR (95% CI)**	**p**	**OR (9 CI)**	**p**
Age of the boy’s mother								
Old	2225	4.6						
Young	151	8.1	1.8 (1.2–2.8)	0.005	1.0 (0.6–1.9)	0.940		
Age of the boy’s father								
Old	2269	4.8						
Young	149	11.4	2.6 (1.5–4.4)	<0.001	2.0 (0.98–4.2)	0.059	1.9 (1.1–3.4)	0.029
Educational level of the boy’s mother								
High	760	2.4						
Low	1789	6.2	2.7 (1.6–4.5)	<0.001	1.9 (1.1–3.4)	0.024	2.2 (1.3–3.8)	0.003
Educational level of the boy’s father								
High	509	2.6						
Low	1833	5.9	2.4 (1.3–4.3)	0.003	1.6 (0.8–3.0)	0.187		
Family structure								
Intact	2182	5.0						
Non-intact	436	5.3	1.1 (0.7–1.7)	0.776				
School performance								
Average or above	2241	4.9						
Below average	425	7.1	1.5 (0.98–2.3)	0.064	0.9 (0.5–1.5)	0.734		
Parent and teacher report of psychopathology (SD)								
Conduct problems (3.0)	2604	-	1.5 (1.3–1.7)	<0.001	1.4 (1.1–1.8)	0.004	1.4 (1.2–1.6)	<0.001
Hyperactivity (2.4)	2587	-	1.3 (1.1–1.5)	<0.001	1.0 (0.8–1.2)	0.810		
Emotional problems (1.9)	2597	-	0.9 (0.8–1.1)	0.366				
Child report of psychopathology								
CDI total score (5.9)	2651	-	1.3 (1.1–1.5)	<0.001	1.2 (1.004–1.4)	0.045	1.2 (0.99–1.4)	0.067
Police register information*								
No criminal offenses	2101	4.3			-			
1-2 offenses	401	7.5	1.8 (1.2–2.7)	0.008	-		1.3 (0.8–2.1)	0.296
3-5 offenses	108	5.6	1.3 (0.6–3.0)	0.546	-		0.7 (0.2–1.9)	0.425
>5 offenses	111	14.4	3.7 (2.1–6.6)	<0.001	-		2.6 (1.3–5.2)	0.008

OR = odds ratio, CI = confidence interval. The variables associated with becoming a young father at significance level p < 0.1 were chosen to the Multipredictor model 1 which included the childhood variables. The variables which remained significant at level p < 0.1 in model 1 were chosen to the model 2 which included the variable on adolescent criminal offenses. OR's > 1 indicate increased likelihood of becoming a young father. The odds ratios for continuous variables (related to psychopathology) were calculated for the change in one SD unit. *Overall p-value was <0.001 in the single predictor model and 0.032 in the multipredictor model.

When the variables, which were associated with becoming a young father at significance level p < 0.1 in the single predictor analysis, were included in the Multipredictor model 1, mother’s low educational level, parent and teacher report of conduct problems and self-report of depressive symptoms remained significant.

To examine whether the association between psychosocial risk factors in childhood and becoming a young father is explained by antisocial behavior in adolescence, Multipredictor model 2 with the variable on adolescent criminal offences was conducted. Of the childhood variables, father’s young age, mother’s low educational level and parent- and teacher-report of conduct problems remained significant. Having more than five criminal offenses in the police register during adolescence remained significant as well.

When two-way interactions between childhood psychopathology variables and family variables were studied, it was found that there are statistically significant interactions between parent and teacher report of emotional problems and father’s educational level (p = 0.032), parent and teacher report of emotional problems and mother’s age (p = 0.016) and parent and teacher report of conduct problems and mother’s educational level (p = 0.045). This indicates that the association between emotional problems and becoming a young father was stronger among boys with a low-educated father or young mother and the association between conduct problems and becoming a young father was stronger among boys with a low-educated mother.

## Discussion

As expected, the proportion of young fathers in the Finnish general population was found to be rather low (5.3%). Boys who had had psychosocial problems in childhood were more likely to become fathers than their peers. The likelihood was increased for those who were born to a young father themselves and for those who have a mother with a low educational level. A particularly interesting finding is that boys, who had had conduct problems at age eight, had an increased risk of becoming a young father, independently of antisocial behavior in adolescence. On the other hand, boys who had repeatedly committed criminal offenses in adolescence, were more likely to become young fathers, largely regardless of their childhood background.

It is known from a previous study on the same cohort that childhood conduct problems are strongly associated with committing offenses in adolescence [23]. In this study, criminality did not mediate the association between childhood conduct problems and becoming a young father. It is possible that different components of childhood conduct problems are associated with criminal activity than with becoming a young father [24]. It may also be that childhood conduct problems and adolescent antisocial behavior are differently related to personality traits such as sensation-seeking or impulsivity, or to contextual factors such as parental monitoring, which are possible mediating factors [25,26]. Miller-Johson et al. [15], whose study showed similar results, suggested that a possible explanatory factor is peer social status. In their study, childhood aggression on one hand and adolescent substance use and deviant peer involvement on the other hand were independent predictors of becoming a young father. The risk of becoming a young father, however, was not increased for boys, who were rejected in their peer group. They hypothesized that boys, who are aggressive in childhood, but not rejected, may become more central members of deviant peer groups and become involved in risky sexual behavior.

The association between self-report of depressive symptoms in childhood and becoming a father was significant before controlling for antisocial behavior in adolescence and close to significant after antisocial behavior was included. This is in contrast with previous studies, which, however, have assessed depressive symptoms in adolescence [8-10,14,18]. Previous studies, which have used the same sample have shown an association between boys’ self-report of depressive symptoms at age eight and low sense of coherence as well as smoking at age 18 [27,28]. These factors may be associated with adolescent risk behavior, but more studies would be needed to confirm the hypothesis.

The finding that sons of young fathers have an increased likelihood of becoming young fathers themselves has also been shown in previous studies [3,29]. Very little is known about possible mechanisms. It has been suggested that the association between parent’s and child’s young age at the time of becoming a parent could be explained by socialization, which refers to attitudes and values which are favorable toward early childbearing, lack of social control in the family and family’s financial and/or marital instability, but most studies have focused on mothers and daughters [30]. In this study, the association became weaker when other family-related factors and childhood psychiatric symptoms were included in the analysis. This suggests that the association between being born to a young father and becoming a young father may be partly explained by adverse childhood environment or psychiatric problems. After all, it may be that in certain communities becoming a father at young age does not break prevailing norms and the intergenerational transmission is explained by cultural factors. An example in Finland is the Laestadian minority, a group of people who belong to a revivalist movement. They are generally opposed to contraception, have high value for family life, high fertility rates and young age at first birth [31].

Low maternal educational level was also associated with becoming a young father. It is known that associations between low socioeconomic status of a family affect children’s health and social outcomes through various family- and community level factors such as stressful life situations, poor access to services, health-compromising life style and adverse neighborhood characteristics [32]. It may be that these factors also increase the likelihood of behavior which leads to becoming a young father. Another possibility is that boys whose mother has a low education have later a low socioeconomic status themselves and are more willing to have a child than for example their peers with higher educational expectations.

The findings of this study have implications in the fields of prevention and parental support. While many boys who become fathers at a young age may be well prepared and have strong social support, there are others who may become fathers unintentionally, lack support and have poor parenting skills. The existing interventions, which aim at supporting young families, are often planned for mothers and do not adequately address the needs of different types of fathers [33]. The boys who have had a high level of conduct problems or who are involved in antisocial behavior in adolescence, are likely to face particular challenges. It is known that young fathers with a history of conduct problems, are likely to be engaged in interpartner violence [34], to spend less time with their children [35] and to have problems in parenting [36]. It has also been shown that parental conduct problems increase the risk of conduct problems among offspring through both genetic and environmental mechanisms [37]. The special needs of boys with a history of conduct problems and/or involvement in risk behavior in adolescence, should be taken into account when providing young parents with psychosocial support and when aiming at improved parenting skills.

It is probable that postponing the timing of becoming a father is sometimes beneficial. When planning sexual health education programs, which aim at reducing adolescents’ risky sexual behavior and unintended pregnancies, it is important, first of all, to acknowledge that boys in general may have different needs than girls [33,38]. In addition, it should be noted that boys, who score high on sensation-seeking and impulsive decision-making, may need interventions different from conventional classroom curricula based on rational decision-making [26]. It should also be studied whether there are any barriers to use of contraception or sexual health services for boys with antisocial tendencies. Early prevention would be preferable, but not much is known about effective methods. In a review on prevention programs of early pregnancy it was shown that girls from disadvantaged families who have participated childhood or youth interventions focusing for example on social and educational support, social skills training or community activities, are less likely to become pregnant in adolescence [39]. However, no statistically significant association has been shown between the participation of boys and their partners’ pregnancies [39]. It is not either known if the prevention or early intervention of conduct problems would decrease the number of young fathers.

There are some limitations in this study. It is possible that the number of young fathers in the sample is underestimated, because not all of them may be registered in the Population Information System. However, this number is likely to be small. In a nationwide Finnish study on autism (FIPS-A, which includes around 5,000 autistic children and 20,000 controls) [40], 98.3% of the children born in 1987–2005 had a registered father. On the other hand, there are probably registered fathers, who are not in reality the biological ones, but there is no official estimate of this figure. Psychiatric symptoms were measured only once. Furthermore, there was no information on other childhood factors such as participants’ cultural background, their religious orientation or stressful life events. Nor was it known whether they were in a permanent relationship and whether becoming a father was a wanted and planned event. Information on criminal offenses should be interpreted with caution, because it is possible that some of the boys had become fathers before committing crimes and reverse causality cannot be ruled out. However, most boys (63%) became fathers after they had turned 20 years. In addition, it is likely that at least those who have committed more than five offenses have started committing them before becoming a father. It would have been interesting to see whether the childhood factors predict differently becoming a father in adolescence versus young adulthood, but the number of young fathers was too small for this kind of analysis.

## Conclusions

This study showed that childhood conduct problems and family-related factors as well as frequent adolescent criminal behavior have an independent association with becoming a young father. However, a more thorough knowledge of the mediating factors and of the interactions between individual and environmental factors would help to give us a better understanding of the possible mechanisms. It is important to include boys in studies which focus on the predictors of becoming a young parent, as well as in prevention and intervention studies.

## Competing interests

The authors declare that they have no competing interests.

## Author’s contributions

VL contributed to the designing of the study and the interpretation of the data, and drafted the manuscript. AS contributed to the designing of the study, the interpretation of the data and the revision of the manuscript, and supervised the study. LS contributed to the designing of the statistical analyses, the interpretation of the data and the revision of the manuscript, and conducted all the statistical analyses. HH contributed to the designing of the statistical analyses, the interpretation of the data and the revision of the manuscript. TT, KK and FA contributed to the collection of the baseline data, the interpretation of the data and the revision of the manuscript. All authors read and approved the final manuscript.

## Pre-publication history

The pre-publication history for this paper can be accessed here:

http://www.biomedcentral.com/1471-2458/12/560/prepub
